# Study on Water Wash Pretreatment and Al-Si Additives to Relieve the Sintering Behavior of Fungus Bran Combustion Ash

**DOI:** 10.3390/molecules29194675

**Published:** 2024-10-01

**Authors:** Dan Wang, Yu Wang, Weinan Xiao, Shengjie Guo, Shuai Guo, Yan Zhao

**Affiliations:** 1 College of Civil Engineering and Water Resources, Heilongjiang Bayi Agricultural University, Daqing 163319, China; wangd@byau.edu.cn (D.W.); sjguo@byau.edu.cn (S.G.); 2 School of Energy and Power Engineering, Northeast Electric Power University, Jilin 132012, China; yuwang1029@foxmail.com (Y.W.); xiaoweinan99@163.com (W.X.); 3 Shenyang Academy of Environmental Sciences, Shenyang 110167, China

**Keywords:** biomass combustion utilization, pretreatment, additives, ash fusion problems

## Abstract

This study focuses on the sintering phenomenon that easily occurs during the direct combustion of molded fuel made from fungus bran (FB). To investigate the key factors influencing sintering, experiments are designed and conducted using a muffle furnace and a high-temperature drop furnace. The experimental results show that the combustion temperature is the primary factor triggering the sintering phenomenon. To effectively mitigate this issue, this study proposes two improvement strategies: water washing pretreatment and the use of additives. The analysis shows that water washing pretreatment effectively removes K and Mg elements, with the removal rates increasing as the washing temperature and time increase. Specifically, the removal rate of K ranges from 37.68% to 55.91%, and that of Mg ranges from 33.16% to 58.52%. Water washing pretreatment also reduces the degree of sintering; at 1400 °C, the TSF (tendency to slag formation) of the fuel increases by 25–40% after pretreatment, with a greater increases observed at higher washing temperatures and longer durations. Kaolin, used as an additive, significantly raises the ash melting point of FB and alleviates sintering, while P_2_O_5_ exacerbates it. Increasing the proportion of kaolin does not significantly enhance the TSF of high-temperature ash, but raising the P_2_O_5_ content from 5% to 10% lowers the TSF by 10–20% at the corresponding temperature.

## 1. Introduction

Given the gradual depletion of coal resources and the growing challenges of environmental protection, the development and utilization of biomass are increasingly becoming focal points of attention. Biomass energy, with its abundant sources, vast reserves, low nitrogen and sulfur contents, and CO_2_ neutrality, has demonstrated great potential and promising prospects as a renewable energy source [1,2,3]. However, the high content of alkali metals (such as K and Na), alkaline earth metals (such as Ca and Mg), and halogen elements (such as Cl and I) in biomass fuels presents significant challenges for its application [4]. During combustion, these elements easily form ash particles, leading to severe sintering and fouling issues [5]. In particular, when the concentration of alkali components is high, they can form high-viscosity, low-melting-point compounds. As these compounds accumulate, they create a hard slag layer that significantly impedes the effectiveness of traditional soot-blowing systems [6,7]. Additionally, the high halogen content in some biomass can release acidic gases during combustion, which can corrode the metal components of combustion equipment. It also emits toxic gases (such as HCl and Cl_2_), posing potential risks to both the environment and human health.

Researchers aim to classify biomass fuels using various indicators to predict their sintering tendencies. Hossein et al. [8] evaluated various sintering prediction indices for biomass fuels from the literature over the past decade, such as the acid-to-base ratio, sintering index, and agglomeration index. Through experiments involving treatments like water washing and acid washing of fuels such as rice husks and sawdust, they found that traditional indices were inadequate due to incomplete element considerations or significant errors. They recommended using improved indices like (K + Na)/P and (K + Na)/(Ca + Mg). Vasileiadou et al. [9] considered the fuel’s calorific value and ash content, proposing a modified index to optimize commonly used prediction parameters. They defined a range for the improved parameters to characterize sintering tendencies and quantified ash sintering strength. By integrating these parameters, they developed a comprehensive evaluation score for ash’s sintering tendencies to differentiate between the sintering strengths of various fuels.

The low ash melting temperature of biomass fuels affects boiler safety, leading researchers to focus on improving their combustion characteristics and addressing issues related to reducing sintering tendencies. Fan et al. [10] studied the combustion characteristics of potassium-rich cotton stalks and found that their ash deformation temperature is low, being around 1260 °C, making them prone to sintering. Adding high-Al-Si coal ash improved the degree of sintering. When the coal ash content increased to 40%, the characteristic temperature of the mixed fuel was higher than that of pure straw ash. Liu et al. [11] found that when rice straw is co-combusted with coal gangue, increasing the proportion of coal gangue can alleviate ash sintering. Specifically, at a ratio of 40–60%, the alkali metals in the ash combine with Al-Si salts to form compounds with high melting temperatures while also reducing CO_2_ emissions. Roberts et al. [12] studied the ash deposition characteristics of two high-potassium biomass ashes (olive cake and whitewood). Their findings from ash fusion experiments suggested that adding an additive (kaolin powder) effectively increases the fuel’s flow temperature. In sintering strength tests, the use of the additive significantly reduces the sintering of the fuel ash. Ash deposition can be removed using a soot blower.

To address the issues of sintering and caking in biomass fuel combustion, in addition to using additives to increase the ash melting point, fuel pretreatment is also an effective method for mitigating sintering tendencies. Melissari et al. [13] investigated methods to improve the ash problems in seasonal biomass fuels such as grass, straw, and vine shoots. They found that the eutectic salts produced during combustion lower the ash melting point. By water-washing sugarcane particles to reduce the content of elements like K, Na, and Mg, the sintering tendency of the ash was significantly improved. Mlonka-Mędrala et al. [14] studied the ash behavior of corn stalks and miscanthus during combustion. They used water, ammonium acetate, and sulfuric acid solutions for leaching to remove K, Na, Ca, and Mg elements, with K and Na significantly affecting low-temperature melting compounds. Water removed approximately 50% of these elements, ammonium acetate removed an additional 10%, and sulfuric acid solution removed only an additional 2%. Mukhopadhyay et al. [15] investigated methods to improve the combustion performances of biomasses such as tobacco stalks, sugarcane bagasse, and poultry waste. By subjecting these biomasses to low-temperature baking, they produced high-energy-density biochar-like (BC) materials. Although this process increased the sintering tendency, washing the BC to remove ash further enhanced its energy density, thereby optimizing the fuel performance.

In summary, research on biomass fuels has primarily focused on elucidating the causes of sintering during combustion, exploring measures to mitigate sintering phenomena (such as additives and pretreatment methods), and developing methods to predict sintering tendencies. However, when it comes to specific biomass feedstocks, current research efforts are heavily concentrated on algae and certain woody biomasses [16], with relatively fewer studies targeting more common materials like corn stalks, wheat straws, and fungus bran as primary experimental subjects. Moreover, the assessment of sintering tendencies in biomass fuels often relies on coal sintering indices, which can introduce biases when applied to biomass fuels. Compared to modifying combustion equipment, directly treating biomass fuels is a more economical and straightforward approach. Therefore, this research project aims to investigate the sintering issues and potential improvements in the practical combustion process of formed fuel made from fungus bran. Using a muffle furnace and a high-temperature drop tube furnace as experimental platforms, this study will employ a combined theoretical and experimental approach to delve into the ash sintering problem. Subsequently, it will analyze the specific causes of sintering during the combustion of fungus bran pellets and propose effective mitigation measures.

## 2. Results and Discussion

### 2.1. The Impact of Water Washing Pretreatment on Fuel Ash Behavior

#### 2.1.1. Analysis of Water Washing Pretreatment (<100 °C) Experiments

##### The Impact of Water Washing Pretreatment on Fuel Properties

After water washing pretreatment, the FB was burned and ashed in a muffle furnace at 600 °C. The ash analysis results are shown in Table 1. An analysis of the ash composition of the raw FB revealed that CaO has the highest proportion (61.23%) due to the presence of quicklime, which has a certain antibacterial effect. Fe_2_O_3_ is the next highest (6.37%). The acidic oxides SiO_2_ and Al_2_O_3_ are present in lower amounts, at 3.66% and 1.14%, respectively. Based on experimental observations that acidic oxides increase the melting temperature and basic oxides decrease it [17,18,19], it is inferred that the melting temperature of this fuel is relatively high. The degree of sintering is defined using a combination of the acid-to-base ratio (B/A), deposit viscosity (Sr), and fouling factor (Fu) indices. Specific standards are provided in Table 2. The specific calculation methods for these evaluation parameters are detailed in Equations (1)–(4). In Equations (1)–(3), the basic oxides Fe_2_O_3_, CaO, MgO, Na_2_O, and K_2_O, as well as the acidic oxides SiO_2_, Al_2_O_3_, P_2_O_5_, Fe_2_O_3_, and MgO, represent the mass percentages (%) of the corresponding components in the ash samples. In Equation (4), m_f_ denotes the mass (g) of the slag sample that passes through the sieve, while m_0_ represents the total mass (g) of the slag sample. According to the standards in Table 2 and based on XRF testing and formula evaluations, the B/A ratio (12.33) and Sr value (0.049) of the raw FB are both in the high-sintering-tendency range, while the Fu value (17.389) is in the moderate-tendency range. This indicates that the molded fuel made from FB has a high sintering tendency and poses a usage risk.

Comparing the original sample, it was found that after water washing, the K_2_O content decreased from 1.22% in the original sample to 0.76% at 20 °C for 5 min, further decreased to 0.63% at 50 °C for 5 min, and dropped to 0.56% at 80 °C for 5 min. The removal rate of alkali metals in the FB increases with temperature. However, the duration of washing has a minimal effect on removal efficiency. For instance, at 20 °C, the K_2_O content remained at 0.76% for 5, 10, and 20 min. At 50 °C, the K_2_O content was 0.63%, 0.69%, and 0.68% for different durations, and at 80 °C, it was 0.56%, 0.54%, and 0.58%, respectively. The water washing pretreatment shows similar removal effects for SO_3_ and MgO as it does for K_2_O, but the contents of SiO_2_ and Al_2_O_3_ increase. Additionally, P_2_O_5_ content increases compared to the original sample. However, the Cl content decreases as the temperature rises.

(1) $$B/A=\frac{{\mathrm{Fe}}_{2}O_{3}+\mathrm{CaO}+\mathrm{MgO}+{\mathrm{Na}}_{2}O+K_{2}O}{{\mathrm{SiO}}_{2}+{\mathrm{Al}}_{2}O_{3}+{{\mathrm{TiO}}_{2}+P}_{2}O_{5}}$$

(2) $$\mathrm{Sr}=\frac{{\mathrm{SiO}}_{2}}{{\mathrm{SiO}}_{2}+{\mathrm{Fe}}_{2}O_{3}+\mathrm{CaO}+\mathrm{MgO}}$$

(3) $$\mathrm{Fu}=\frac{B}{A}\left({\mathrm{Na}}_{2}O+K_{2}O\right)$$

(4) $$\mathrm{TSF}=\frac{m_{f}}{m_{0}}\times 100\%$$

**Figure 1:**
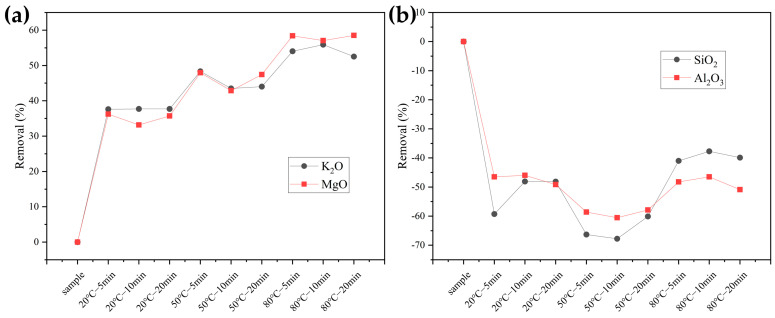
The removal of ash components in samples after pretreatment (**a**) K_2_O and MgO; (**b**) SiO_2_ and Al_2_O_3._

As shown in Figure 1a, it illustrates the removal rates of K_2_O and MgO. It can be observed that the removal of both elements is significant during the pretreatment process, with similar removal rates. At 20 °C, the removal rates for K are 37.62%, 37.78%, and 37.80%, while for Mg they are 36.22%, 33.16%, and 35.71% at the same temperature. At 50 °C, the removal rates for K are 48.36%, 43.52%, and 44.02%, whereas for Mg they are 47.96%, 42.86%, and 47.45%. At 80 °C, the removal rate of Mg is slightly higher than that of K, with K removal rates of 54.02%, 55.91%, and 52.54% and Mg removal rates of 58.42%, 57.04%, and 58.52%. Overall, increasing the pretreatment temperature enhances the removal of both K and Mg.

Figure 1b shows the removal rates of SiO_2_ and Al_2_O_3_. Since the mass density of both elements increases after water washing pretreatment, the *y*-axis is negative (a negative *y*-axis removal rate indicates an increase in the element). The growth rates for Si at 20 °C are 59.29%, 48.09%, and 48.11%, while the corresponding growth rates for Al at the same temperature are 46.49%, 45.96%, and 49.12%. At 50 °C, the growth rates for Si are 66.34%, 67.76%, and 60.11%, and for Al, they are 58.61%, 60.53%, and 57.89%. At 80 °C, the growth rates for Si are 40.98%, 37.71%, and 39.89%, while for Al they are 48.25%, 46.49%, and 50.88%. The growth rates of Si and Al increase from 20 °C to 50 °C with rising temperature but decrease at 80 °C, falling even below the growth rates at 20 °C. Additionally, the changes in Al are relatively stable and less affected by washing, whereas Si shows significant variation and is more influenced by washing.

In summary, pretreatment time has minimal impact on the removal/increase rates of elements, whereas changes in pretreatment temperature significantly affect these rates.

Based on the parameters for evaluating coal combustion performance (B/A, Sr, Fu), the relevant data for the samples pretreated with water are substituted into Equations (1)–(3), and the results are shown in Table 3. It can be observed that the B/A ratio decreases from 12.33 in the raw sample to 7.80 (under the 20 °C—5 min condition). It then slightly increases to 8.41 (20 °C—10 min) and is 8.34 at 20 °C—20 min. Under the 50 °C—10 min condition, the B/A ratio reaches its lowest value of 7.43, subsequently increasing to 7.87 (50 °C—20 min) and then rising and stabilizing at around 8.5 under the later pretreatment conditions.

The Sr value is relatively low, increasing from 0.050 in the raw sample to 0.08 (20 °C—5 min). It then slightly decreases to 0.07 at 20 °C—20 min. The Sr value reaches 0.08 at 50 °C—10 min, and for the three samples pretreated at 80 °C, the Sr value remains relatively stable at 0.07.

Changes in the Fu values of the fuels after water washing pretreatment are as follows: The Fu value decreases from 17.39 in the raw sample to 7.18 (20 °C—5 min). It then increases slightly to 7.82 and 7.75 at 20 °C—10 min and 20 °C—20 min, respectively, before decreasing again. At 50 °C, the Fu values are 6.70, 6.61, and 7.26 for the 5 min, 10 min, and 20 min conditions, respectively. At 80 °C, the Fu values further decrease to 6.23, 5.94, and 5.98 for the 5 min, 10 min, and 20 min conditions, respectively, with the minimum value of 5.94 achieved at 80 °C—10 min.

In summary, after water washing pretreatment, the fuel removes a certain proportion of alkali metals and other elements prone to forming low-melting-point compounds. This results in a decrease in the B/A and Fu values and an increase in the Sr value, all of which indicate a reduction in the risks associated with these three sintering parameters. Based on the predicted results from the elemental proportions, water washing pretreatment has a positive effect.

##### The Impact of Water Washing Pretreatment on Fuel Sintering Degree

**Figure 2:**
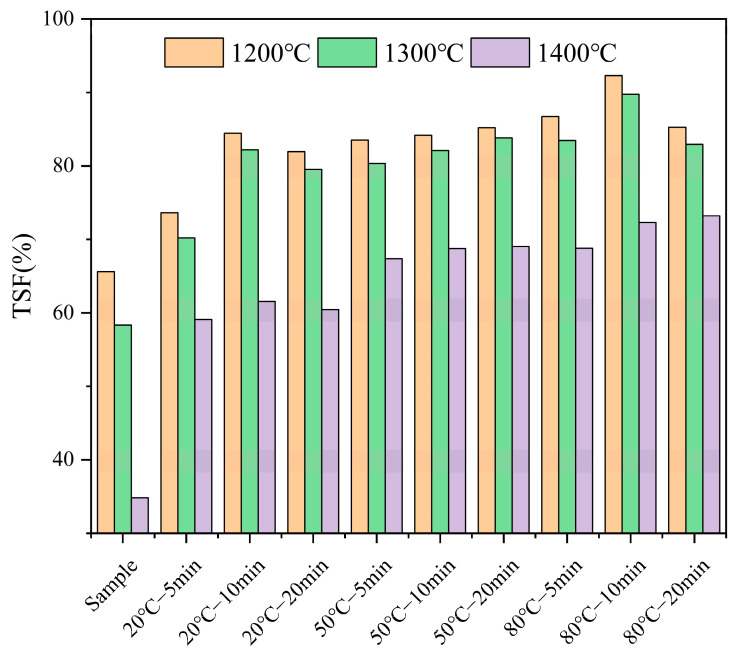
The sintering degree of each fuel after water washing pretreatment.

To investigate the impact of water washing pretreatment on the high-temperature sintering of fuel made from FB, high-temperature settling furnace tests were conducted, considering only temperature variables (1200 °C, 1300 °C, 1400 °C) to assess the sintering conditions. The sintering degree was quantified using TSF values. The results, shown in Figure 2, compared the sintering severity of the raw sample with that of the pretreated fuel.

As shown in Figure 2, the sintering degree of the raw FB increases with the rise in combustion temperature, with the TSF value dropping significantly. The TSF decreased from 65.61% at 1200 °C to 58.33% at 1300 °C, and nearly halves to 34.82% at 1400 °C. Water washing mitigated the sintering issue of FB to some extent. In the combustion environment at 1200 °C, the TSF increased by 8%, 19%, and 16% after pretreatment at 20 °C for 5, 10, and 20 min, respectively. Pretreatment at 50 °C for 5, 10, and 20 min further increased the TSF by 18%, 19%, and 20%, respectively. At 80 °C, the TSF increased by 21%, 27%, and 20% after 5, 10, and 20 min of pretreatment, respectively, with the two pretreatment conditions (except for 80 °C—10 min) showing improvement effects on TSF similar to those of the three pretreatments at 50 °C. It can be observed that both pretreatment time and temperature are effective at mitigating the sintering issue in a high-temperature environment of 1200 °C.

At 1300 °C, water washing pretreatment significantly improved the sintering issue of the FB ash. Under pretreatment at 20 °C for 5, 10, and 20 min, the TSF increased by 12%, 24%, and 21% compared to the original TSF of 58.33%. Pretreatment at 50 °C for 5, 10, and 20 min resulted in increases of 22%, 24%, and 26%, respectively. At 80 °C, the TSF increased by 25%, 31%, and 27% after 5, 10, and 20 min of pretreatment, respectively. Except for the 80 °C—10 min condition, the improvement effects on TSF from the other two 80 °C pretreatments were similar to those observed in the three 50 °C pretreatments.

At the high temperature of 1400 °C, the sintering issue became more pronounced, with the TSF of the raw sample being 34.82%. Water washing pretreatment effectively alleviated this problem, with TSF increases of 24%, 26%, and 25% recorded after pretreatment at 20 °C for 5, 10, and 20 min, respectively. Pretreatment at 50 °C for 5, 10, and 20 min resulted in increases of 32%, 34%, and 33%, respectively. At 80 °C, the TSF increased by 34%, 37%, and 38% after 5, 10, and 20 min of pretreatment, respectively.

In summary, water washing pretreatment improved the sintering issue of FB in high-temperature combustion environments, with the effect being particularly significant at 1400 °C. The degree of sintering decreased as the pretreatment temperature and time increased. However, the TSF improvement at 80 °C was not always significantly greater than at 50 °C, indicating that the effectiveness of the pretreatment may have limits and become more apparent under higher sintering risks.

#### 2.1.2. Analysis of Water Washing Pretreatment Experiments (>100 °C)

##### Effect of Water Washing Pretreatment on Fuel Properties

**Figure 3:**
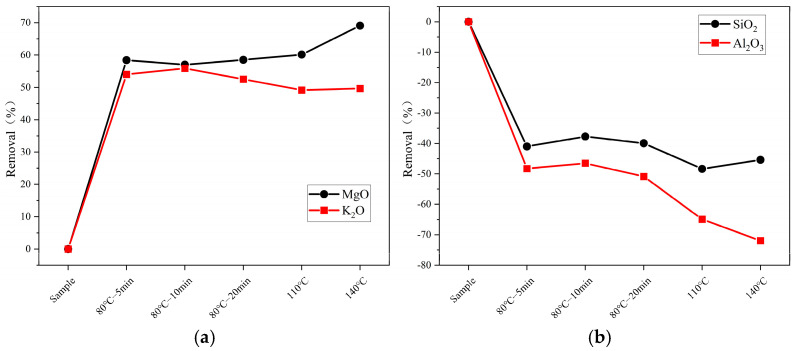
The removal of ash components in samples subjected to high-temperature pretreatment (**a**) K_2_O and MgO; (**b**) SiO_2_ and Al_2_O_3._

An analysis of the ash components of FB after high-temperature pretreatment is shown in Table 4. It can be observed that, as mentioned in previous studies, the removal rates of K and Mg increase significantly with higher water washing pretreatment temperatures. Conversely, the removal rates of Si and Al decrease. The removal rates of these elements after high-temperature pretreatment are illustrated in Figure 3.

Figure 3 shows the changes in ash content of elements such as K, Mg, Si, and Al under different temperatures (80 °C for 5, 10, and 20 min; 100 °C; 140 °C; and a control group) during pretreatment. In (a), the figure shows that the removal rate of K remains stable at 49% at 100 °C and 140 °C, which is about 3–5% lower than that at 80 °C. In contrast, the removal rate of Mg increases with temperature, reaching 60% at 100 °C and 69% at 140 °C. In (b), the figure illustrates the increases in the Si and Al elements. It is evident that the increase rate of both elements grows with temperature. The increase rates of Si are 48% and 45% at 100 °C and 140 °C, respectively, approximately 8% and 5% higher than those at 80 °C. The increase in Al is more pronounced, with removal rates of 65% and 72% at 100 °C and 140 °C, respectively, about 15% and 72% higher than at 80 °C. Additionally, Table 1 and Table 3 reveal that the removal rates of certain elements increase with temperature during conventional temperature pretreatment (20 °C, 50 °C, 80 °C). However, atypical behavior is observed at high temperatures (110 °C and 140 °C), as seen with Cl and S elements. This is suspected to be related to differences in the seal quality of the heating vessels (beakers versus hydrothermal reactors), which affect the volatilization behavior of the elements.

Table 5 shows the predicted sintering tendencies of the fuel after high-temperature pretreatment. The results show that the B/A ratios at 110 °C and 140 °C are 8.1 and 8.4, respectively, which are slightly lower than the average B/A ratio of 8.5 at 80 °C, indicating a tendency towards lower sintering. Conversely, the Fu values at 110 °C and 140 °C are 6.6 and 6.7, respectively, compared to an average Fu value of 6.0 at 80 °C. This suggests that samples subjected to high-temperature pretreatment have slightly higher Fu values, indicating a tendency towards higher sintering. The Sr values at 110 °C and 140 °C are 0.075 and 0.074, respectively, which are 0.004 and 0.003 higher than the average Sr value of 0.071 at 80 °C. This indicates a slightly weaker sintering tendency for samples after high-temperature pretreatment. For samples subjected to high-temperature pretreatment, although B/A and Sr tend to indicate reduced sintering, Fu suggests an increased sintering tendency. However, the fluctuations in these values are minimal, indicating that the impact of high-temperature pretreatment on the sintering tendency of the samples is not significantly different from that of conventional pretreatment.

##### Effect of Water Washing Pretreatment on Fuel Sintering Degree

**Figure 4:**
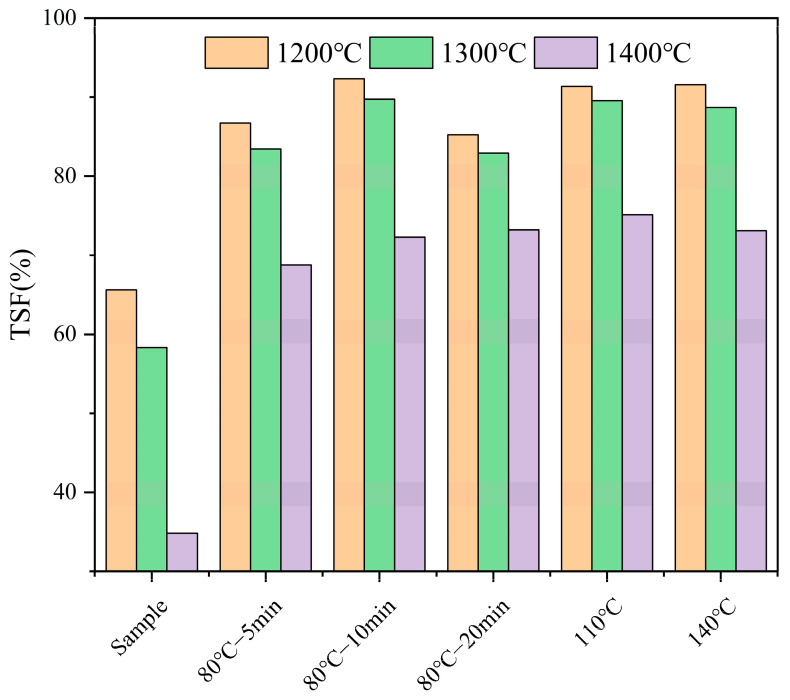
The sintering degrees of various fuels after high-temperature pretreatment.

The analysis of the effect of high-temperature pretreatment on the ash components and sintering tendency of FB fuel shows that compared to 80 °C pretreatment, 110 °C and 140 °C pretreatment do not significantly enhance the sintering tendency. Figure 4 shows the sintering degree of ash samples after high-temperature combustion. At 1200 °C, the ash TSF for samples pretreated at 110 °C and 140 °C are 91.37% and 91.58%, respectively, which are approximately 3% higher than the average TSF of 88% for the 80 °C pretreatment groups at the same combustion temperature. The TSF values for 110 °C and 140 °C are nearly the same. At 1300 °C, the ash TSF for samples pretreated at 110 °C and 140 °C are 89.56% and 88.71%, respectively, about 4% higher than the average TSF of 85% for the 80 °C pretreatment groups at the same combustion temperature. However, at the higher combustion temperature of 1400 °C, the TSF values for samples pretreated at 110 °C and 140 °C are 75.13% and 73.24%, respectively, which are almost the same as the average TSF value of 72% for the 80 °C pretreatment groups at this combustion temperature. At all three combustion temperatures, the TSF values for samples pretreated at 110 °C and 140 °C are quite similar, suggesting that the effect of water washing pretreatment temperature on the fuel’s sintering degree may be approaching its limit.

### 2.2. Effect of Additives on Fuel Ash Behavior

#### 2.2.1. Effect of Additives on Fuel Properties

Table 6 shows the changes in the ash components of the fuel after adding different proportions of additives. The relevant data for the original samples and samples with additives are used in Equations (1)–(3), and the results of the sintering predictions are shown in Table 7. The results show that after mixing with additives, the B/A ratio of the original FB significantly decreases from 12.33, indicating a tendency towards moderate sintering. When 5% and 10% kaolin are added, the B/A ratio of the fuel decreases to 6.44 and 4.21, respectively, showing a significant reduction. Similarly, the additions of 5% and 10% P_2_O_5_ also lead to notable decreases in the B/A ratio compared to the original FB. At the same time, the extents of the decreases in the B/A ratio increase with the addition proportions of the additives. Since Fu is derived from B/A, the trends in Fu and B/A with increasing additive types and proportions are similar.

The changes in Sr values show that all additive groups have higher Sr values compared to the original FB (0.0499), indicating a tendency towards moderate sintering. After mixing with 5% and 10% kaolin, the Sr values increase to 0.084 and 0.1231, respectively. This increase is greater than that observed with kaolin in the water washing pretreatment groups. In contrast, the Sr value remains unchanged when P_2_O_5_ is added, as the calculation does not account for P_2_O_5_. Therefore, the increase in P_2_O_5_ content does not affect the predicted sintering tendency based on Sr values.

The introduction of additives can improve the parameters predicting the sintering tendency of the fuel, and the effect becomes more pronounced with increasing additive proportions. Kaolin significantly increases the Sr value, while the effect of P_2_O_5_ cannot be analyzed due to the limitations of the predictive parameters, which do not fully represent the variable ash compositions of biomass fuels [21,22,23].

#### 2.2.2. Mechanism of Additives on the Sintering Degree of Fuels

##### Influence of Additives on Sintering Degree

**Figure 5:**
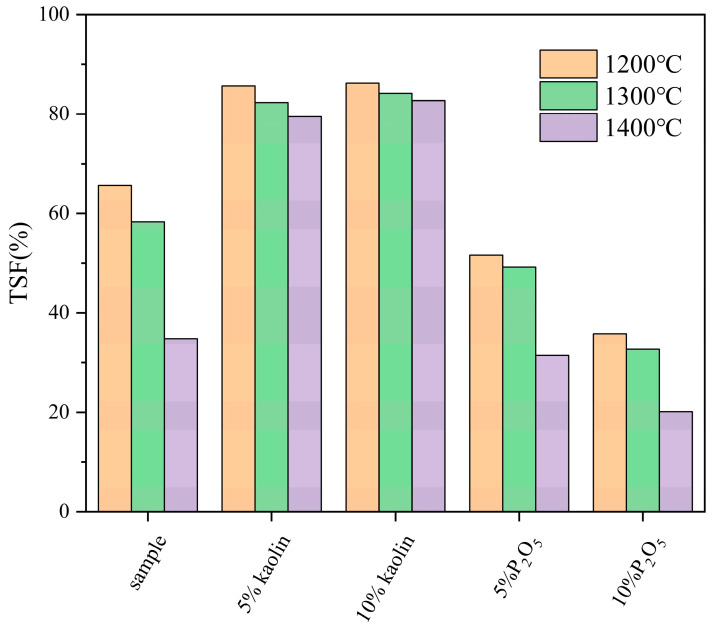
The sintering degree of original FB and fuel mixed with additives.

To investigate the effects of additives on the high-temperature sintering behavior of fuels, samples of FB mixed with kaolin and P_2_O_5_ were tested using a high-temperature settling furnace. Ash samples were prepared at 1200 °C, 1300 °C, and 1400 °C, and their ash fusion temperatures (TSFs) were measured. The results are shown in Figure 5. As the environmental temperature increases, the TSF of the fuel decreases. This trend is observed in untreated samples, those mixed with additives, and pretreated samples (whether at room temperature or high temperature) of FB. The TSF values of the untreated FB significantly decreased at 1200 °C, 1300 °C, and 1400 °C, with corresponding TSF values of 65.61%, 58.33%, and 34.82%, respectively. However, after mixing with kaolin, the TSF significantly increased at the same temperatures. With the addition of 5% kaolin powder, the TSF value at 1200 °C was 85.66%, an increase of 20.05% compared to the original sample; at 1300 °C, it was 82.32%, an increase of 23.99%; and at 1400 °C, it was 79.51%, an increase of 44.69%, close to 45%. Adding 10% kaolin resulted in even higher TSF improvements at different temperatures. At 1200 °C, the TSF value was 86.23%, a 20.62% increase compared to the original sample; at 1300 °C, it was 84.17%, an increase of 25.84%; and at 1400 °C, the TSF was 82.73%, an increase of 47.91%, close to 50%. This indicates that kaolin, as an additive, can effectively alleviate the sintering behavior of the fuel. The proportion of kaolin additive (5% and 10%) has a relatively minor impact on the TSF of the fuel. The difference in enhancement at the same temperature is only between 2% and 3%, with the difference being nearly negligible at 1200 °C, being just 0.5%.

Unlike kaolin additives, which improve the sintering issue of FB, P_2_O_5_ additives exacerbate this problem. Under reaction conditions at 1200 °C, the TSF value of the fuel with 5% P_2_O_5_ additive is 51.61%, a reduction of 13.99% compared to the original sample. At 1300 °C, the TSF value of the mixed fuel is 49.21%, a decrease of 9.12% compared to the original sample. At 1400 °C, the TSF of the mixed fuel is 31.47%, a reduction of 3.35% compared to the original sample. With a 10% P_2_O_5_ additive, the TSF value of the mixed fuel further decreases. At 1200 °C, 1300 °C, and 1400 °C, the TSF values are 35.79%, 32.73%, and 20.14%, respectively. Compared to the original FB sample, the TSF values decrease by 29.81%, 25.60%, and 14.68% at the same temperatures. Compared to the original FB, the addition of P_2_O_5_ significantly reduces the TSF values of the fuel at the same reaction temperatures, indicating an exacerbation of the sintering degree of the ash samples. Since TSF represents the ratio of the mass of ash that can pass through a sieve to the total mass of the ash sample, a lower TSF value indicates a higher degree of sintering.

In summary, kaolin and P_2_O_5_ additives have different effects on the ash melting behavior of FB: kaolin improves sintering, though the improvement diminishes with higher proportions, while P_2_O_5_ worsens sintering, with the degree of sintering significantly increasing as the proportion rises.

##### Mechanism of Kaolin Additive on the Sintering of FB

**Figure 6:**
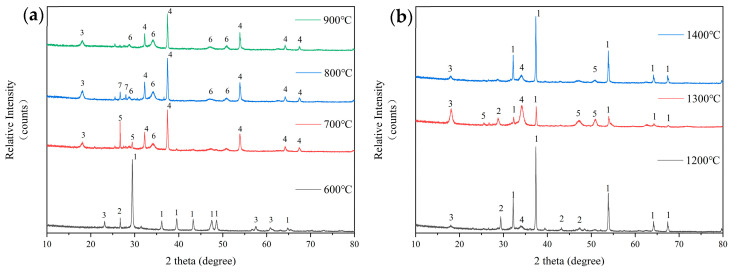
Analysis of ash composition from the combustion of original FB at low temperatures (**a**): 1—Calcite-CaCO_3_; 2—Hydrobiotite (K-Mg-Fe-Si-Al-Fe-O-OH); 3—Illite-KAl_3_Si_3_O_10_(OH)_2_; 4—Lime, syn-CaO; 5—Okenite-CaSi_2_O_5_; 6—Portlandite-Ca(OH)_2_; 7—Sodium Silicate-Na_2_SiO_3_. An analysis of ash composition from the combustion of original FB at high temperatures (**b**): 1—Lime, syn–CaO; 2—Calcium Sulfate-CaSO_4_; 3—Illite-KAl_2_Si_3_AlO_10_(OH)_2_; 4—Palmerite-K_4_Al_4_P_6_O_23_(2Al_2_O_3_·2K_2_O·3P_2_O_5_); 5—Okenite-CaSi_2_O_5_.

The XRD analysis results of the original FB are shown in Figure 6a,b. Figure 6a shows the analysis of ash composition from the original FB after combustion at 600 °C to 900 °C. At 600 °C, the primary component of the ash is calcite (CaCO_3_), which is attributed to the quicklime (CaO) added during cultivation to prevent moisture. This component dominates during combustion. It also contains small amounts of illite (KAl_3_Si_3_O_10_(OH)_2_) and hydrous biotite (K-Mg-Fe-Si-Al-Fe-O-OH). As the combustion temperature rises to 700 °C, the decomposition of carbonates causes the ash’s main component to shift to lime (CaO), which combines with other elements to form wollastonite (CaSi_2_O_5_), portlandite (Ca(OH)_2_), and a residual amount of illite (KAl_3_Si_3_O_10_(OH)_2_). When the temperature rises to 800 °C, wollastonite (CaSi_2_O_5_) significantly decreases while lime (CaO) increases, and an intermediate product, sodium metasilicate (Na_2_SiO_3_), is formed. When the combustion temperature reaches 900 °C, the ash is primarily composed of CaO, with small amounts composed of illite and portlandite.

Figure 6b shows the composition analysis of ash samples from the original FB prepared in a high-temperature settling furnace. At 1200 °C, the ash is primarily composed of CaO, with small amounts of CaSO_4_, illite (KAl_3_Si_3_O_10_(OH)_2_), and palmierite (2Al_2_O_3_·2K_2_O·3P_2_O_5_). At 1300 °C, the ash is still primarily composed of CaO. Compared to the composition at 1200 °C, it now also contains new amounts of wollastonite (CaSi_2_O_5_) and an increased quantity of palmierite. At 1400 °C, in addition to CaO being the main component of the ash, CaSO_4_ disappears, and both wollastonite (CaSi_2_O_5_) and illite significantly decrease.

From 700 °C onwards, CaO becomes the main component of the FB ash. At high temperatures, the ash sinters, and while CaO remains the primary component, the relative amounts of secondary components decrease with increasing temperature. This indicates that the primary reason for the sintering of FB ash at high temperatures is the approach of the melting point of the main ash component CaO (approximately 1500 °C).

**Figure 7:**
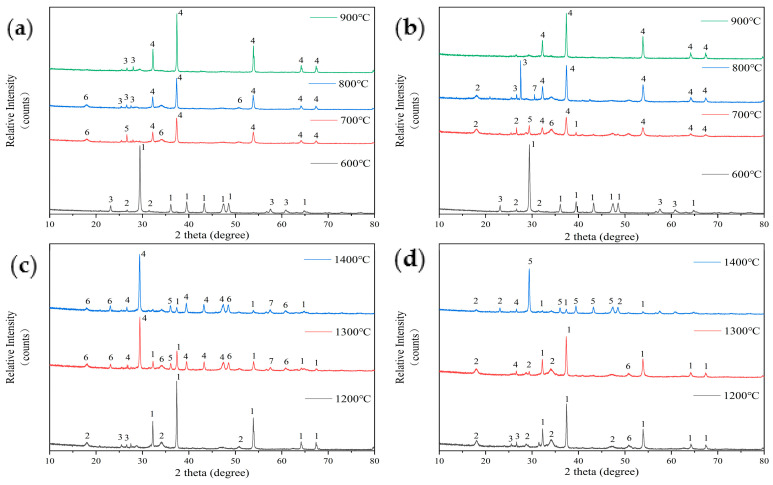
An analysis of ash composition in low-temperature ash of FB with 5% kaolin addition (**a**): 1—Calcite-CaCO_3_; 2—Hydrobiotite-(K-Mg-Fe-Si-Al-Fe-O-OH); 3—Illite-KAl_2_Si_3_AlO_10_(OH)_2_; 4—Lime, syn-CaO; 5—Lazurite (Sodalite-Ultramarine)-(Na-Ca-Al-Si-O-S-Cl); 6—Portlandite-Ca(OH)_2_. An analysis of ash composition in low-temperature ash of FB with 10% kaolin addition (**b**): 1—Calcite-CaCO_3_; 2—Lepidolite (Mica)-(K-Mg-Fe-Al-Si-O); 3—Illite-KAl_2_Si_3_AlO_10_(OH)_2_; 4—Lime, syn-CaO; 5—Calcium Sulfate-CaSO_4_; 6—Hydrobiotite-(K-Mg-Fe-Si-Al-Fe-O-OH); 7—Okenite-CaSi_2_O_5_; An analysis of ash composition in high-temperature ash of FB with 5% kaolin addition (**c**): 1—Lime, syn-CaO; 2—Lazurite (Sodalite-Ultramarine)-(Na-Ca-Al-Si-O-S-Cl); 3—Calcium Sulfate-CaSO_4_; 4—Okenite-CaSi_2_O_5_; 5—Calcite-CaCO_3_; 6—Illite-KAl_2_Si_3_AlO_10_(OH)_2_; 7—Nephrite-Ca(Mg,Fe)_3_(SiO_3_)_4_. An analysis of ash composition in high-temperature ash of FB with 10% kaolin addition (**d**): 1—Lime, syn-CaO; 2—Illite-KAl_2_Si_3_AlO_10_(OH)_2_; 3—Lepidolite (Mica)-(K-Mg-Fe-Al-Si-O); 4—Riversideite-Ca_2_SiO_3_; 5—Okenite-CaSi_2_O_5_, 6—Lazurite (Sodalite-Ultramarine)-(Na-Ca-Al-Si-O-S-Cl).

The introduction of kaolin additives significantly alters the mass proportion of elements in the fuel, thereby affecting the mineral phase composition of the ash. Figure 7 shows the analysis results of ash composition after the addition of kaolin. Figure 7a shows that at 600 °C, the ash composition of FB with 5% kaolin addition is similar to that of the original sample, with calcite (CaCO_3_) being the main component and small amounts of illite (KAl_3_Si_3_O_10_(OH)_2_) and hydrous biotite (K-Mg-Fe-Si-Al-Fe-O-OH) also being present. The difference is that the additive reduces the amount of hydrous biotite and slightly increases the illite content, consistent with the trend of kaolin addition enhancing aluminosilicate products. However, when the temperature rises to 700 °C, the ash contains lapis lazuli (Na-Ca-Al-Si-O-S-Cl), indicating that kaolin has fixed the volatile Na and Cl elements. At this temperature, Ca(OH)_2_ also appears, and the main component of the ash shifts to lime (CaO). By 800 °C, lapis lazuli disappears, and illite (KAl_2_Si_3_AlO_10_(OH)_2_) appears. At 900 °C, Ca(OH)_2_ disappears, and the diffraction peak intensity of lime (CaO) increases.

Figure 7b shows the analysis of the low-temperature ash composition of FB with 10% kaolin addition. At 600 °C, the ash composition is the same as that with 5% kaolin addition. Once the temperature reaches 700 °C, the main component of the ash transforms into CaO, along with a small amount of CaSO_4_ and water-bearing biotite (K-Mg-Fe-Si-Al-Fe-O-OH). This composition differs from that of ash with 5% added kaolin, which does not contain lazurite (Na-Ca-Al-Si-O-S-Cl) or Ca(OH)_2_. Instead, it contains complex compounds such as lithium mica (K-Mg-Fe-Al-Si-O) and water-bearing biotite (K-Mg-Fe-Si-Al-Fe-O-OH) formed by the combination of multiple elements with Al and Si. As the temperature rises to 800 °C, the main component of the ash, CaO, further increases. Lithium mica and water-bearing biotite disappear, and illite (KAl_2_Si_3_AlO_10_(OH)_2_) and feldspar (CaSi_2_O_5_) appear. Beyond 800 °C, no components other than CaO are detected in the ash.

Comparing Figure 6 and Figure 7, the addition of kaolin as an Al-Si-based additive results in a significant increase in Al-Si compounds in the ash from FB. These compounds often include elements such as K, Na, or Cl, or a combination of them, such as illite, lithium mica, and water-bearing biotite. These compounds gradually disappear between 700 °C and 900 °C, leading to a delay in the release of alkali metal halide compounds, which were expected to begin volatilizing at 600 °C [24].

Figure 7c shows the results of the sintering tendency analysis of ash components from FB with 5% kaolin additive after combustion in a high-temperature settling furnace. At 1200 °C, the main component of the ash is lime (CaO), with a small amount of lazurite (Na-Ca-Al-Si-O-S-Cl) and CaSO_4_. After mixing in 5% kaolin, the composition of the ash is similar to that of the original sample. At 1300 °C, the main component of the ash transforms into calcium silicate (CaSi_2_O_5_), with a reduced amount of CaO. It also contains small amounts of illite (KAl_2_Si_3_AlO_10_(OH)_2_) and jadeite (Ca(Mg,Fe)_3_(SiO_3_)_4_). At 1400 °C, the main component of the ash remains calcium silicate, with an increased diffraction peak intensity. The rest of the composition is similar to that at 1300 °C, except that the CaO content has further decreased. After adding kaolin, new high-melting-point Al-Si compounds (such as calcium silicate, illite, and jadeite) appear in the ash [12,25], indicating an improvement in the sintering issues of FB.

Figure 7d shows that at 1200 °C, the ash from fuel with 10% kaolin additive primarily consists of CaO, along with small amounts of illite (KAl_2_Si_3_AlO_10_(OH)_2_), lithium mica (K-Mg-Fe-Al-Si-O), and lazurite (Na-Ca-Al-Si-O-S-Cl). In contrast, the ash from the original FB and the fuel with 5% kaolin at 1200 °C contains more Al-Si compounds. At 1300 °C, the main component of the ash remains lime (CaO), with lithium mica disappearing and calcium silicate (Ca_2_SiO_3_) appearing. The chemical composition of the ash is similar to that of ash, having the same kaolin additive ratio at 1200 °C. At 1400 °C, the main component of the ash becomes calcium silicate (CaSi_2_O_5_), accompanied by small amounts of illite and calcium metasilicate.

With the addition of 5% or 10% kaolin, at 1400 °C, the main components of the ash are calcium silicate (CaSi_2_O_5_) and small amounts of Al-Si compounds. In contrast, the original FB ash primarily consists of CaO (with a melting point of about 1500 °C) at both 1300 °C and 1400 °C, leading to sintering behavior. The addition of kaolin effectively increases the fuel’s temperature of softening (TSF) and reduces the degree of sintering because the melting point of CaSi_2_O_5_ in the high-temperature ash approaches 2000 °C, significantly raising the melting point of the ash.

##### The Mechanism of P_2_O_5_ Additive on the Sintering of FB

**Figure 8:**
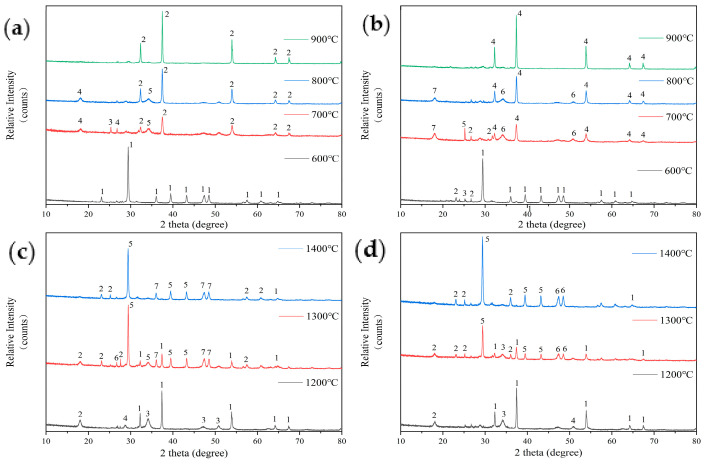
An analysis of the ash composition of FB with 5% P_2_O_5_ addition at low temperatures (**a**): 1—Calcite-CaCO_3_; 2—Lime, syn-CaO; 3—Iron(III) Chamosite-(Fe-Mg-Al-Si-Al-O-OH); 4—Lazurite (Sodalite-Ultramarine)-(Na-Ca-Al-Si-O-S-Cl); 5—Palmerite-K_4_Al_4_P_6_O_23_(2Al_2_O_3_·2K_2_O·3P_2_O_5_). An analysis of the ash composition of FB with 10% P_2_O_5_ addition at low temperatures (**b**): 1—Calcite-CaCO_3_; 2—Illite-KAl_2_Si_3_AlO_10_(OH)_2_; 3—Kaolinite-Al_2_Si_2_O_5_(OH)_4_; 4—Lime, syn-CaO; 5—Iron(III) Chamosite-(Fe-Mg-Al-Si-Al-O-OH); 6—Portlandite-Ca(OH)_2_; 7—Lazurite (Sodalite-Ultramarine)-(Na-Ca-Al-Si-O-S-Cl). An analysis of the ash composition of FB with 5% P_2_O_5_ addition at high temperatures (**c**): 1—Lime, syn–CaO; 2—Illite-KAl_2_Si_3_AlO_10_(OH)_2_; 3—Palmerite-K_4_Al_4_P_6_O_23_(2Al_2_O_3_·2K_2_O·3P_2_O_5_); 4—Okenite-CaSi_2_O_5_; 5—Brushite-CaHPO_4_; 6—Hydrobiotite-(K-Mg-Fe-Si-Al-Fe-O-OH); 7—Calcite-CaCO_3_. An analysis of the ash composition of FB with 10% P_2_O_5_ addition at high temperatures (**d**): 1—Lime, syn–CaO; 2—Illite-KAl_2_Si_3_AlO_10_(OH)_2_; 3—Palmerite-K_4_Al_4_P_6_O_23_(2Al_2_O_3_·2K_2_O·3P_2_O_5_); 4—Natrolite-Na_2_Al_2_Si_3_O_10_; 5—Brushite-CaHPO_4_; 6—Calcite-CaCO_3_.

The results of the composition analysis of the ash from FB after mixing with the phosphorus-based additive P_2_O_5_ are shown in Figure 8. Figure 8a shows that the main component of the low-temperature ash from FB with 5% P_2_O_5_ addition at 600 °C is calcium carbonate (CaCO_3_). This is consistent with the composition of the ash from earlier test samples after low-temperature combustion. After 700 °C, the main component of the ash changes to lime (CaO), and phosphoaluminate (2Al_2_O_3_·2K_2_O·3P_2_O_5_) appears. This indicates that the introduction of the phosphorus-based additive has initiated new thermochemical reactions. At 800 °C and 900 °C, the ash primarily consists of CaO, with only small amounts of glauconite (Fe-Mg-Al-Si-Al-O-OH) and lazurite (Na-Ca-Al-Si-O-S-Cl) being present at 800 °C.

Figure 8b shows the composition analysis of the ash with 10% P_2_O_5_ additive. At 600 °C, the main component is still calcium carbonate (CaCO_3_), along with small amounts of illite (KAl_2_Si_3_AlO_10_(OH)_2_) and kaolinite (Al_2_Si_2_O_5_(OH)_4_). When the temperature reaches 700 °C, the main component of the ash becomes lime (CaO), and glauconite (Fe-Mg-Al-Si-Al-O-OH) and lazurite (Na-Ca-Al-Si-O-S-Cl) are also formed. These components are consistent with those found when 5% P_2_O_5_ is added at the same temperature. However, at a 10% P_2_O_5_ ratio, calcium hydroxide (Ca(OH)_2_) is also detected. At 800 °C, the amount of calcium hydroxide (Ca(OH)_2_) decreases. Apart from this, the composition of the ash is consistent with that of the fuel ash with 5% P_2_O_5_ addition at 900 °C.

Both P_2_O_5_ and kaolin additives alter the proportions of certain elements, but they have a limited impact on the crystalline phase composition of low-temperature ash below 1000 °C. At 600 °C and 900 °C, the main components of the ash are calcium carbonate and calcium oxide, respectively. At 700 °C and 800 °C, special products appear, such as water-bearing biotite and illite with complex Al-Si compounds when kaolin is added and phosphoaluminate when P_2_O_5_ is introduced. Therefore, addressing the sintering issues of FB requires focusing on the thermochemical reactions during the high-temperature combustion process.

After adding P_2_O_5_, the sintering of FB during high-temperature combustion is intensified, indicating that P_2_O_5_ affects the combustion chemical reactions and results in the formation of low-melting-point ash. Figure 8c shows that at 1200 °C, the main component of the ash from FB with 5% P_2_O_5_ addition is lime (CaO), along with phosphoaluminate (2Al_2_O_3_·2K_2_O·3P_2_O_5_) and illite (KAl_2_Si_3_AlO_10_(OH)_2_), as well as a small amount of calcium silicate (CaSi_2_O_5_). Aside from the presence of phosphoaluminate, the composition is similar to that of the original FB ash. As the temperature increases to 1300 °C, the main component of the ash shifts to calcium phosphate (CaHPO_4_), with a significant reduction in CaO content, and the presence of water-bearing biotite (K-Mg-Fe-Si-Al-Fe-O-OH). As the temperature rises further to 1400 °C, the CaO content in the ash decreases even more, and the amount of calcium phosphate increases correspondingly.

Figure 8d shows the composition of the ash from wood ear fungus cultivation residue with 10% P_2_O_5_ addition. At 1200 °C, the main component of the ash is CaO, with small amounts of illite (KAl_2_Si_3_AlO_10_(OH)_2_) and phosphoaluminate (2Al_2_O_3_·2K_2_O·3P_2_O_5_). This composition is similar to that with 5% P_2_O_5_ addition, but it also includes an additional presence of calcium silicate (CaSi_2_O_5_). As the temperature rises to 1300 °C, the main component of the ash similarly changes to calcium phosphate (CaHPO_4_). The previously dominant CaO decreases, and the diffraction peaks of illite and phosphoaluminate become weaker. At 1400 °C, the ash composition is the same as that with 5% P_2_O_5_ addition, with calcium phosphate (CaHPO_4_) being the main component.

After adding the phosphorus-based additive P_2_O_5_, the high-temperature sintering of FB is intensified (evidenced by a decrease in the temperature of softening (TSF)). Comparing the ash composition after high-temperature combustion of the original FB, which primarily consists of CaO, to that with the phosphorus-based additive, which transforms into CaHPO_4_, reveals that the high-temperature ash sample contains hard but brittle “spheres” that were only observed after the high-temperature reaction with this additive. It is speculated that these spheres are primarily composed of CaHPO_4_ or its derivatives, which leads to a significant decrease in the TSF value of the FB after adding P_2_O_5_.

## 3. Materials and Methods

### 3.1. Materials

We used FB recovered from the fungus bran in Huangsongdian, Jilin City, Jilin Province, as biomass pellet fuel. Due to the weathering and surface damage of the received formed particles, they were first subjected to crushing. After crushing, a 100-mesh sieve was used to collect the powder within the size range of 0–150 microns. This powder was then dried in an oven at 105 °C for 24 h. Finally, 1.0 ± 0.1 g of the dried fungus bran powder was weighed and re-pressed using a manual hydraulic press to form particles with uniform density and an undamaged surface. These particles were then stored for subsequent experiments. Ultimate analysis of the samples was performed using an SDLA 718 industrial analyzer (Sandy, China) and proximate analysis was conducted with an EA 3000 elemental analyzer (Euro Vector, Italy) according to GB/T 30732-2014 [26] and GB/T 30733-2014 [27], respectively. The mass percentage of sulfur (S) in the samples was measured using an SDS350 infrared sulfur analyzer (Sandy, China) according to GB/T 25214-2010 [28], while the mass percentage of oxygen (O) was determined by the difference method. The calorific value was measured using an SDC311 oxygen bomb calorimeter (Sandy, China). The measurement results are shown in Table 8.

### 3.2. Ash Sample Preparation

The combustion performance of FB pellet fuel was analyzed through combustion tests using a muffle furnace (Yanre Co., Ltd., Harbin, China). Undamaged samples (15 ± 0.5 g) were selected, and different final temperatures (600–900 °C) were set to investigate the combustion characteristics. A temperature of 600 °C was used as the baseline, aiming to minimize the release of alkali metals and halogens in the ash. Therefore, the ash produced by burning FB at 600 °C was considered the original ash. During the heating process, the temperature was increased from room temperature to 200 °C at a rate of 3 °C/min and from 200 °C to 550 °C at a rate of 6 °C/min. Between 240 °C and 400 °C, volatile matter was released, producing a significant amount of smoke. The heating rate had to be controlled, and the furnace door had to be opened at the appropriate time to allow the volatile matter to burn completely. After complete combustion, the furnace door was closed, and the temperature was maintained at 550 °C for 3 h to ensure a complete reaction and stable ash composition. The ash was then cooled, ground, sieved, and properly stored for subsequent experimental analysis.

### 3.3. Selection of Additives and Sample Preparation

Our analysis of the ash composition of FB pellet fuel revealed that it belongs to the high-calcium type. The additive used was a common Al-Si additive, kaolin powder [10,29]. Al-Si additives can form aluminosilicates during high-temperature chemical reactions, and these aluminosilicates generally have high melting points [30]. Additionally, phosphorus-based additives are also used in other biomass materials [31,32]. Therefore, kaolin was selected as the Al-Si-based additive and P_2_O_5_ as the phosphorus-based additive. The reagents were first ground and sieved using a 100-mesh screen to collect powders in the 0–150 µm size range. These powders were then mixed with FB powder in mass ratios of 0%, 5%, and 10%, respectively. The mixtures were subsequently pressed into briquettes for further ashing and combustion tests.

### 3.4. Analytical Methods

#### 3.4.1. High-Temperature Slagging Experiments of FB Pellet Fuel

**Figure 9:**
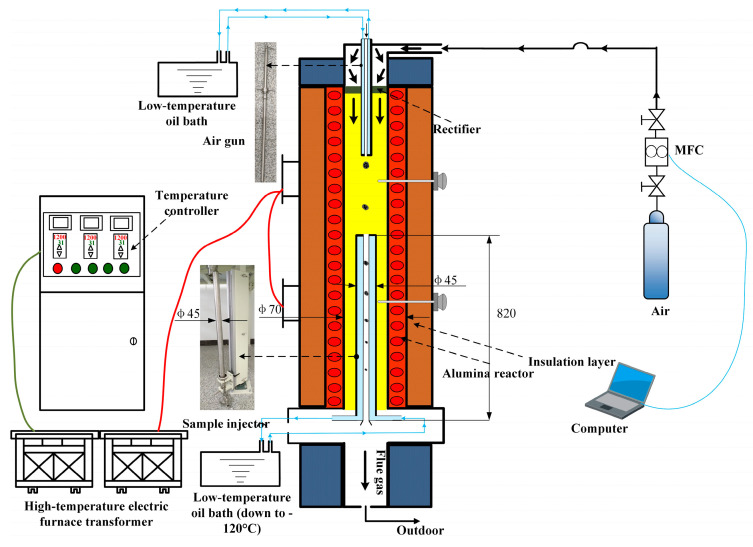
A system diagram of the high-temperature settling furnace experimental setup.

High-temperature slagging experiments of FB pellet fuel were conducted in a high-temperature settling furnace (DTF, Yanre Co., Ltd., Harbin, China). The experimental setup is shown in Figure 9. This study combined traditional coal combustion performance evaluation parameters, such as the acid-to-base ratio (B/A), deposit viscosity (Sr), and fouling index (Fu), with the innovative ‘sieving method’ (TSF) to assess the sintering degree of high-temperature slag samples from FB pellet fuel.

The DTF experimental system primarily comprised a gas distribution system, a preheater, the reactor body, a sampling system, and a water cooling system. Among these components, the reactor section as constructed of high-purity corundum tubing (with a length of 1.5 m and an inner diameter of 60 mm), capable of conducting isothermal tests up to 1500 °C. This system not only simulated actual working conditions but also met a diverse range of experimental requirements. Specifically, the gas distribution system enabled precise control over the gas flow rates necessary for the experiment, ensuring experimental accuracy. The preheater ensured that the gas temperature entering the reactor reached a preset value, thereby enhancing experimental stability. Furthermore, the water cooling system safeguarded the proper operation of experimental equipment, preventing damage caused by high temperatures.

For the high-temperature slag formation experiments of formed fuel from fungus bran, the final temperatures were set at 1200 °C, 1300 °C, and 1400 °C [19,22]. The DTF experimental platform was heated at a rate of 200 °C/2 h until reaching the target temperature. Upon reaching the target temperature, the test gas (air) was introduced at a flow rate of 2000 mL/min, and the system was maintained without any further operation for 30 min to stabilize the internal temperature field. For each test, 1.0 ± 0.1 g of ash samples was weighed and placed in a high-temperature-resistant alumina crucible, which was then secured on a mullite base. The crucible, along with the sample pusher at the bottom of the drop tube furnace, was inserted into the isothermal zone of the furnace. After reaching the desired residence time, the sample pusher was retracted, and the crucible was allowed to cool to room temperature before being removed. The high-temperature slag samples of the formed fuel from fungus bran were collected from the crucible, recorded, and stored for subsequent testing and analysis.

#### 3.4.2. Water Washing Pretreatment Experimental Scheme

To remove elements in FB that easily form low-melting-point substances, a solid-to-liquid ratio of 1 g:15 mL was used to mix fungus residue powder with deionized water. The pretreatment process involved combinations of different temperatures and times, with specific conditions detailed in Table 9. After pretreatment, the solid and liquid phases were separated by vacuum filtration. The solid portion was then dried in a constant-temperature drying oven at 105 °C for over 12 h to remove external moisture. After drying, the material was ground and sieved using a 100-mesh screen to collect powder in the 0–150 µm range. A sample of 1.0 ± 0.1 g of this powder was then re-formed using a manual hydraulic press.

#### 3.4.3. XRF and XRD Testing

XRF analysis of the ash chemical composition of FB pellet fuel was conducted using an S8 TIGER X-ray fluorescence spectrometer (Bruker, City of Saarbrucken, Germany). Additionally, XRD testing was performed on the ash and slag samples prepared from burning the pellet fuel in a muffle furnace and a high-temperature settling furnace. The XRD analysis was carried out with a D8 VENTURE/QUEST X-ray diffractometer (Bruker, Germany), using Cu/Kα radiation (40 kV, 40 mA) for crystal analysis. The scanning range was 5–90° with a scanning speed of 5°/min. The results were analyzed using MDI Jade 6.5 software for phase identification.

## 4. Conclusions

This study explores the mechanism behind the sintering phenomenon occurring during the direct combustion of FB fuel and examines the improvement of this issue through water washing pretreatment and the addition of mixed additives. The conclusions are as follows:

(1) The FB fuel is prone to sintering at high temperatures. At 1300 °C, the ash content begins to aggregate and contract, and sintering becomes significant at 1400 °C. Adjusting the combustion residence time revealed that the degree of sintering is similar at 5 min and 10 min, indicating that under these conditions, temperature has a stronger influence on sintering behavior than residence time.
(2) Water washing pretreatment significantly raises the fuel’s characteristic temperatures, particularly HT and FT, which exceed 1500 °C. DT and HT also increase by nearly 100 °C each. With the optimal pretreatment (20 °C for 20 min), DT reaches 1373 °C, and ST reaches 1412 °C. The sintering prediction indices B/A and Sr also increase, indicating an improvement in sintering behavior.
(3) Water washing pretreatment effectively removes potassium (K) and magnesium (Mg) elements, with the removal efficiency increasing with higher temperatures. Conversely, the contents of silicon (Si) and aluminum (Al) elements increase, and the extent of this increase also rises with temperature. The pretreatment temperature has a greater impact on the changes in element composition in FB than the pretreatment time. TSF indicates the degree of sintering, which significantly increases after pretreatment: it rises by approximately 10% at 1200 °C, 20% at 1300 °C, and 30% at 1400 °C. However, both temperature and time contribute to an increase in TSF, but once the temperature reaches a certain range, the rate of increase begins to diminish. This suggests that there is a limit to the improvement in the sintering behavior of the FB fuel due to pretreatment.
(4) The impact of additives on the combustion of FB is limited, only slightly affecting the mass changes at different stages. Both additives improve the predicted indicators, but since the Sr value calculation does not involve phosphorus (P) elements, the effect of adding P_2_O_5_ on combustion behavior cannot be predicted. Kaolin helps to improve sintering, while P_2_O_5_ worsens the degree of sintering. The improvements with 5% and 10% kaolin are similar, while increasing the amount of P_2_O_5_ exacerbates the sintering process.
(5) The main component of the ash from raw FB at 1300 °C and 1400 °C is CaO, which has a melting point of approximately 1500 °C, leading to sintering issues at high temperatures. Kaolin changes the main component of the ash to CaSi_2_O_5_, which has a melting point above 2000 °C, thereby alleviating sintering behavior by raising the melting point. However, when P_2_O_5_ is added, the main component of the ash becomes CaHPO_4_, resulting in an increased degree of sintering in the FB ash samples.

The significance of this research lies in utilizing fungus bran, which would otherwise be landfilled, as a biomass fuel for electricity generation and heating, representing an environmentally friendly and resource-recycling approach. However, its limitations can be summarized by three points:

(1) The excessively long retention time chosen for the water washing pretreatment process fails to clearly elucidate the sequence of removal for different elements;
(2) There are alternative pretreatment methods in industry, such as acid washing or the use of organic solvents, yet this study has not explored their potential improvements in addressing sintering issues associated with biomass fuels;
(3) While predictive indices like B/A were employed in this study, there is a discrepancy between these predictions and actual outcomes, particularly because the most crucial factor affecting sintering, namely combustion temperature, has not been incorporated into such predictive indicators.

## Figures and Tables

**Table 1:** An analysis of ash composition for each sample after water washing pretreatment.

Sample Conditions	CaO	Fe_2_O_3_	SiO_2_	MgO	K_2_O	SO_3_	Al_2_O_3_	P_2_O_5_	Na_2_O	Cl
Sample	61.23	6.38	3.66	1.96	1.22	1.19	1.14	0.96	0.19	0.047
20 °C—5 min	58.31	6.69	5.83	1.25	0.76	1.28	1.67	1.11	0.16	0.048
20 °C—10 min	58.69	6.55	5.42	1.31	0.76	1.45	1.55	1.05	0.17	0.043
20 °C—20 min	59.68	6.64	5.42	1.26	0.76	1.40	1.70	1.10	0.17	0.038
50 °C—5 min	59.65	6.51	4.99	1.02	0.63	0.77	1.58	1.24	0.14	0.030
50 °C—10 min	58.44	6.66	6.14	1.12	0.69	1.33	1.83	1.06	0.20	0.034
50 °C—20 min	59.52	7.03	5.86	1.03	0.68	1.13	1.80	1.05	0.24	0.038
80 °C—5 min	59.86	6.70	5.16	0.82	0.56	0.65	1.69	1.14	0.17	0.036
80 °C—10 min	60.23	6.50	5.04	0.84	0.54	0.62	1.67	1.20	0.15	0.033
80 °C—20 min	59.68	6.40	5.12	0.81	0.58	0.59	1.72	1.18	0.13	0.026

**Table 2:** The fuel sintering tendency evaluation scheme [20].

Evaluation Indicators	Low Tendency	Moderate Tendency	High Tendency
B/A	B/A < 0.5	0.5 < B/A < 1.0	B/A > 1.0
Sr	Sr > 72	65 < Sr < 72	Sr < 65
Fu	Fu < 0.6	0.6 < Fu < 40	Fu > 40

**Table 3:** The predicted sintering tendency of each fuel after water washing pretreatment.

Sample Conditions	B/A	Sr	Fu
Sample	12.33	0.05	17.39
20 °C—5 min	7.80	0.08	7.18
20 °C—10 min	8.41	0.08	7.82
20 °C—20 min	8.34	0.07	7.75
50 °C—5 min	8.70	0.07	6.70
50 °C—10 min	7.43	0.08	6.61
50 °C—20 min	7.87	0.08	7.26
80 °C—5 min	8.52	0.07	6.23
80 °C—10 min	8.63	0.07	5.94
80 °C—20 min	8.43	0.07	5.98

**Table 4:** An analysis of ash components in samples after high-temperature water washing pretreatment.

Sample Conditions	CaO	Fe_2_O_3_	SiO_2_	MgO	K_2_O	SO_3_	Al_2_O_3_	P_2_O_5_	Na_2_O	Cl
sample	61.23	6.38	3.66	1.96	1.22	1.19	1.14	0.96	0.19	0.047
80 °C—5 min	59.86	6.70	5.16	0.82	0.56	0.65	1.69	1.14	0.17	0.036
80 °C—10 min	60.23	6.50	5.04	0.84	0.54	0.62	1.67	1.20	0.15	0.033
80 °C—20 min	59.68	6.40	5.12	0.81	0.58	0.59	1.72	1.18	0.13	0.026
110 °C	59.34	6.62	5.43	0.78	0.62	0.92	1.88	1.03	0.20	0.041
140 °C	59.23	6.41	5.32	0.61	0.61	0.96	1.96	0.72	0.19	0.068

**Table 5:** The sintering tendency of various fuels after high-temperature water washing pretreatment.

Sample Conditions	B/A	Sr	Fu
Sample	12.33	0.05	17.39
80 °C—5 min	8.52	0.07	6.23
80 °C—10 min	8.63	0.07	5.94
80 °C—20 min	8.43	0.07	5.98
110 °C	8.10	0.08	6.64
140 °C	8.40	0.07	6.74

**Table 6:** An analysis of ash components in original fungus bran (FB) and fuels with added additives.

Conditions	CaO	Fe_2_O_3_	SiO_2_	MgO	K_2_O	SO_3_	Al_2_O_3_	P_2_O_5_	Na_2_O	Cl
Sample	61.23	6.38	3.66	1.96	1.22	1.19	1.14	0.96	0.19	0.047
5% kaolin	58.1685	6.05625	6.227	1.862	1.159	1.1305	3.333	0.90725	0.1805	0.04465
10% kaolin	55.107	5.7375	8.794	1.764	1.098	1.071	5.526	0.8595	0.171	0.0423
5% P_2_O_5_	58.1685	6.05625	3.477	1.862	1.159	1.1305	1.083	5.90725	0.1805	0.04465
10% P_2_O_5_	55.107	5.7375	3.294	1.764	1.098	1.071	1.026	10.8595	0.171	0.0423

**Table 7:** The predictive sintering tendency of original and additive fuels.

Sample Conditions	B/A	Sr	Fu
Sample	12.33	0.05	17.39
5% kaolin	6.44	0.09	8.63
10% kaolin	4.21	0.12	5.34
5% P_2_O_5_	6.44	0.05	8.63
10% P_2_O_5_	4.21	0.05	5.34

**Table 8:** The industrial analysis, elemental analysis, and calorific value of FB.

Industrial Analysis/(%, ad)		Elemental Analysis/(%)		Calorific Value/(MJ/kg)
M_ad_	4.23	C	43.81	17.45
A_ad_	10.09	H	5.94	
V_ad_	70.56	O_a_	33.75	
FC_ad_	15.12	N	0.79	
		S	1.36	

Note: O = 100—C—H—N—S—Mad-Aad (%).

**Table 9:** Water washing pretreatment conditions.

Water Washing Pretreatment Conditions			
Temperature	20 °C	50 °C	80 °C
Time	5min	10min	20min

## Data Availability

Data are contained within the article.
